# Proteome response at the edge of protein aggregation

**DOI:** 10.1098/rsob.140221

**Published:** 2015-02-11

**Authors:** Natalia Sanchez de Groot, Ricardo A. Gomes, Anna Villar-Pique, M. Madan Babu, Ana Varela Coelho, Salvador Ventura

**Affiliations:** 1 Medical Research Council Laboratory of Molecular Biology, Francis Crick Avenue, Cambridge CB2 0QH, UK; 2 Instituto de Tecnologia Química e Biológica António Xavier, Universidade Nova de Lisboa, Av. da República, 2780-157 Oeiras, Portugal; 3 Department of Neurodegeneration and Restorative Research, University Medical Center Goettingen, Waldweg 33, Goettingen, Germany; 4 Institut de Biotecnologia i Biomedicina and Departament de Bioquímica i Biologia Molecular, Universitat Autònoma de Barcelona, 08193, Bellaterra (Barcelona), Spain

**Keywords:** protein misfolding, oxidative stress, amyloid-β-peptide, proteomic response

## Abstract

Proteins adopt defined structures and are crucial to most cellular functions. Their misfolding and aggregation is associated with numerous degenerative human disorders such as type II diabetes, Huntington's or Alzheimer's diseases. Here, we aim to understand why cells promote the formation of protein foci. Comparison of two amyloid-β-peptide variants, mostly insoluble but differently recruited by the cell (inclusion body versus diffused), reveals small differences in cell fitness and proteome response. We suggest that the levels of oxidative stress act as a sensor to trigger protein recruitment into foci. Our data support a common cytoplasmic response being able to discern and react to the specific properties of polypeptides.

## 2. Introduction

The formation of aggregates is not restricted to disease-linked proteins, but rather constitutes a generic property of polypeptide chains, hence cells have to deal with protein misfolding and aggregation regularly. As a result, they have evolved a set of tools and strategies for control and defence to limit protein misfolding and aggregation [1–4]. They possess chaperones to assist proteins in folding and protect them during their lifetime [1–3,5,6]. When proteins are not necessary or irreversibly damaged, autophagy and proteasome systems ensure their removal. Molecular chaperones and proteolysis pathways are the principal components of the unfolded protein response (UPR) that operates when dangerous misfolded proteins are detected [1–3,5–7]. The coordinated action of this machinery ensures a tight regulation of protein homeostasis [1–3,5,6,8]. When these control systems become altered, cells begin to malfunction and pathologies may manifest.

Currently, there are still unsolved questions regarding the elements involved in protein quality control (PQC), and the specific mechanisms that modulate protein aggregation *in vivo*, mainly because tracking the fate of a protein in the intracellular milieu is challenging owing to its crowded and complex composition. In this context, yeast has arisen as a powerful model organism to understand not only the PQC machinery but also to address the pathological role of protein aggregation in human disease [9]. As a first attempt to unravel the yeast PQC response against protein misfolding, we recently expressed 20 GFP-fused peptides in yeast, derived from amyloid-β-peptide (Aβ42), that cover a continuous range of aggregation propensities [10,11]. Interestingly, despite most of these peptides being highly insoluble, just some of them are recruited into foci. With this approach, we identified an aggregation propensity threshold above which the cell actively accumulates a protein into foci [11]. Here, we use two proteins from this collection, which are located on either side of the aggregation threshold, to decipher why protein foci are or are not formed in cells. Specifically, we characterize how these two proteins impact on cell fitness and cellular homeostasis. Our results support that the formation of inclusion bodies is an energetically expensive process that protects the cell against harmful effects associated with misfolded proteins, including oxidative stress [12–14]. We suggest that levels of oxidative stress may serve as a trigger for protein recruitment into foci. Overall, the data presented here indicate that the cellular response to protein misfolding is able to discern and accommodate the specific properties of polypeptides (e.g. aggregation propensity).

## 3. Results and discussion

### 3.1. Protein aggregation with enhanced proteolysis

**Figure 1.**
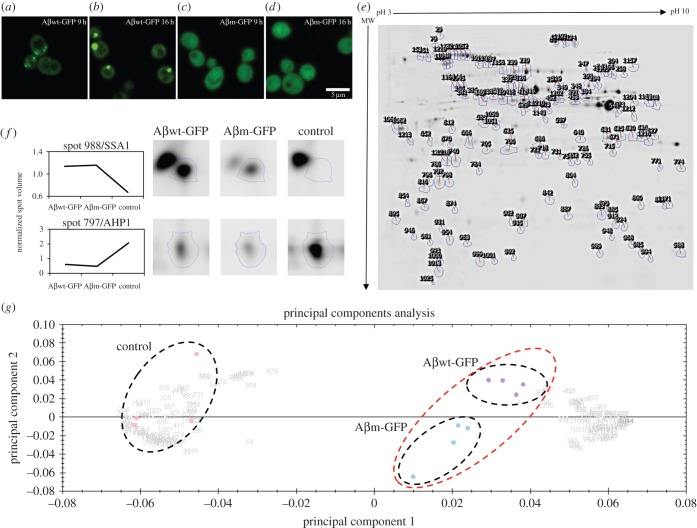
Differential protein expression analysis. (*a*) Aβwt-GFP after 9 and (*b*) 16 h of induction. (*c*) Aβm-GFP after 9 and (*d*) 16 h of induction. (*e*) Representative two-dimensional-DIGE gel image showing the differential spot map. The gel shows those spots with a significant ANOVA value of *p* < 0.05 when comparing the quadruplicates of the three samples analysed (control, Aβwt-GFP and Aβm-GFP). The proteins identified in these spots are listed in the electronic supplementary material, table S2, table S3 and table S5. (*f*) Example of the two main spot volume trends observed: higher abundance (Spot 988/SSA1) or decreased abundance (Spot 797/AHP1) in yeast expressing Aβ variants when compared with the control strain. (*g*) Principal component analysis of the two-dimensional-DIGE results. Each point represents the global expression values for all statistically significant spots of each gel image analysed. Three different groups of gels can be identified (black dashed circles): the control strain (pink), Aβwt-GFP (purple) and Aβm-GFP (blue). However, Aβwt and Aβm are very close (red dashed circles).

The GFP-tagged peptides employed in this work are the Aβ42 wild-type (Aβwt) and the mutant Aβ42 F19D (Aβm), which includes a single substitution by a gatekeeper residue (aspartate) that disrupts a central hydrophobic stretch and reduces the aggregation propensity [10,11,15]. Actually, the presence of gatekeepers (charged residues and proline) flanking aggregation-prone regions is an evolutionary strategy to prevent anomalous protein self-assembly [16,17]. In yeast, Aβwt and Aβm are mostly insoluble (electronic supplementary material, table S1 and figure S1) but exhibit distinct intracellular distributions: inclusion body (Aβwt) versus diffuse (Aβm) (figure 1*a–d*). Thus, according to our recent report, they are located at two sides of an aggregation propensity threshold that determines proteins' intracellular deposition into foci [11]. We analysed the expression and the soluble/insoluble partition of these two polypeptides after 9 h of induction, when multiple foci are formed in Aβwt-GFP (figure 1*a*). Before 9 h their numbers are low, and after that time all foci are recruited in a single big focus, reaching an equilibrium state where no size changes or new foci are observed [15] (figure 1*b*). No foci were detected for Aβm-GFP during the course of the experiment (figure 1*c*–*d*).

Despite the similarity in mRNA levels of Aβwt-GFP and Aβm-GFP, their relative abundances upon translation are significantly different (electronic supplementary material, table S1 and figure S1). Measuring the loss of GFP fluorescence after blocking translation, we observed that Aβwt-GFP fluorescence disappears six times faster than Aβm-GFP, indicating a stronger proteolytic activity acting on the more aggregation-prone variant. This suggests that the process of foci formation transfers the protein to a different degradation pathway [11,14] with enhanced proteolysis that results in a shorter Aβwt-GFP half-life and a higher Aβm-GFP intracellular concentration (electronic supplementary material, table S1 and figure S5). Similar results were obtained in *Escherichia coli*, with an aggregation-prone segment of σ32β also tagged with GFP, in which the presence of different gatekeepers affected cell fitness, not only by modulating the fusion intrinsic aggregation propensity but also by regulating its abundance through a differential activity of the PQC [17].

### 3.2. Aβwt-GFP foci and diffused Aβm-GFP cause similar cell fitness effects

The comparison between Aβwt-GFP and Aβm-GFP expressing cells did not show significant differences in growth rate (electronic supplementary material, table S1 and figure S2). Intriguingly, there is controversy around the consequences of protein aggregation on cell fitness. Drummond and co-workers showed that misfolding and aggregation of YFP and Ura3p variants, containing multiple amino acid substitutions, decrease cell viability [7]. However, Korona and co-workers recently reported no significant correlation between cell fitness and the insoluble fraction of Ade2p mutants [18] and, like our observations with Aβm-GFP, part of Ade2p wild-type (the most soluble variant) is present in the insoluble fraction. Overall, these data suggest that in our system the formation of inclusion bodies probably acts as a protective mechanism or at least a non-toxic one. In fact, several lines of evidence point to the prefibrillar and oligomeric species being the toxic species responsible for the onset of human disorders such as Huntington's or Alzheimer's disease [13,19,20]. These reports suggest that the mature fibrils are much less toxic [20,21] and that the formation of inclusion bodies may act as a detoxifying mechanism against the accumulation of early species [13,14].

### 3.3. A common cytosolic unfolding protein response

**Figure 2.**
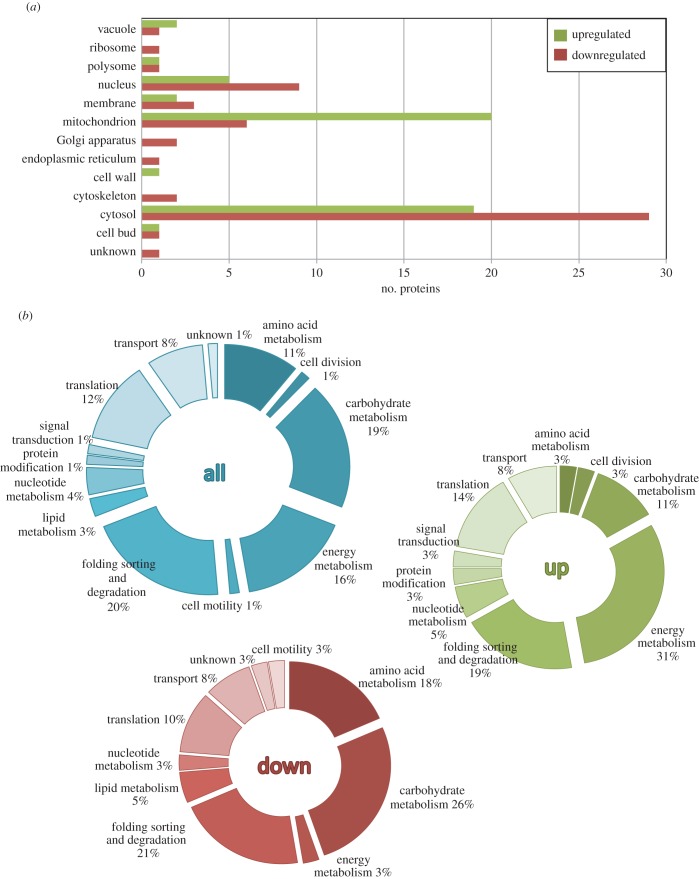
Cellular component distribution and KEGG pathway classification. (*a*) Cellular localization of the proteins upregulated and downregulated in Aβwt-GFP when compared with the control strain. (*b*) Functional classification of all the proteins identified (blue). Proteins downregulated (red) and upregulated (green) in comparison with the control strain.

To analyse the cellular response against the insoluble but aggregating Aβwt-GFP and the insoluble but non-aggregating Aβm-GFP, we performed a comparative two-dimensional-DIGE analysis (figure 1*e*) between cells expressing plasmid without insert (control), Aβwt-GFP or Aβm-GFP. Two different patterns could be identified in more than 95% of the spots analysed: protein abundance in Aβwt-GFP and Aβm-GFP strains either increases (electronic supplementary material, table S2) or decreases (electronic supplementary material, table S3) with respect to the control strain (figure 1*f*). In agreement, principal component analysis of the gel images shows that they can be separated into three groups, but Aβwt-GFP and Aβm-GFP images are closer to each other and distant from the control strain (figure 1*g*). Overall, we conclude that the main part of the cell response to Aβwt-GFP and Aβm-GFP is common, and only 12 proteins are specially adjusted because of them (electronic supplementary material, table S4). The proteins detected in the common response are associated with cytoplasm (65%), mitochondrion (35%) and nucleus (19%; figure 2*a*). According to the gene ontology classification for cellular function obtained from Kyoto Encyclopaedia of Genes and Genomes (KEGG; http://www.genome.jp/kegg/), the proteins detected belong mainly to three categories: protein folding, sorting and degradation (20%), carbohydrate metabolism (19%) and energy metabolism (16%; figure 2*b*). These proteins and categories also overlap with the metabolic and quality control adjustments recently reported by us and other authors upon expression of different heterologous proteins, such as the human transthyretin or a misfolded variant of YFP [7,22] (electronic supplementary material, table S5). These responses share multiple elements with a cytosolic unfolding protein response (UPR-Cyto), such as the upregulation of HSF1 targets (e.g. Ssa1, Ssa2, Hsc82; electronic supplementary material, table S5) and a small ribosomal repression (e.g. RPS21A, RS21B, EFB1) in comparison with the UPR associated with endoplasmic reticulum [7,8,23]. Hence, our results support the existence of a universal response to control cytosolic misfolding in yeast, probably similar to that occurring in mammalian cells, owing to the similarity between large yeast foci and mammalian aggresomes [24].

### 3.4. Aβwt-GFP versus Aβm-GFP: proteome differences

**Figure 3.**
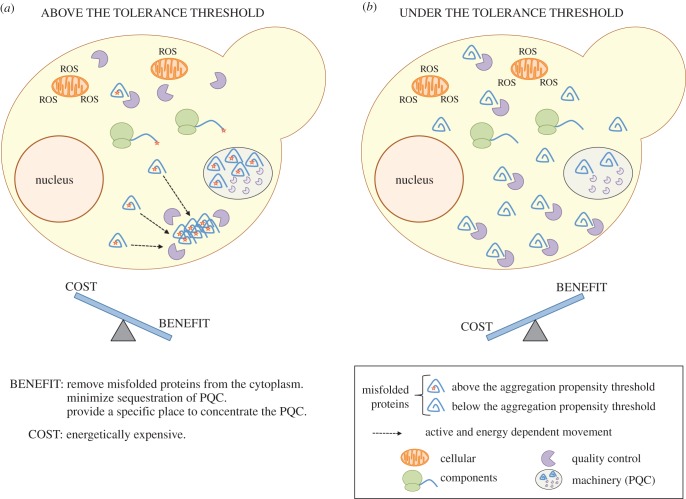
One unfolded protein response: two strategies to control the toxic effects. Model showing the different benefits and costs of accumulating a misfolded protein into foci. Specifically, this diagram shows two proteins, mostly insoluble, but located either side of an aggregation threshold above which the cell actively recruits a protein into foci [11] (*a*). This process could offer benefits but it is also energetically expensive (cost). Under the threshold, the protein remains diffuse through the cytoplasm (*b*). This could favour the formation of harmful interactions that could initiate a cascade of misfolding and oxidative stress. ROS, reactive oxygen species.

Apart from this common response, we detected 12 proteins differentially regulated between Aβwt-GFP and Aβm-GFP (electronic supplementary material, table S4). Surprisingly, despite divergences in protein half-life (electronic supplementary material, figure S2), none are related to proteolytic response. Their absence could be explained with the benefits provided by foci formation: (i) removing the dangerous misfolded protein from the cytoplasm, (ii) minimizing stoichiometric sequestration of PQC components thereby freeing them to assist their normal partners and (iii) accumulating the deleterious misfolded polypeptide into a localized aggregate where the quality control cellular machinery can be concentrated (figure 3). Therefore, the enhanced proteolysis of Aβwt-GFP does not require an extra expression of PQC machinery because the foci formation itself may make it more efficient. Aβwt-GFP has downregulated ARC15, which is associated with actin polymerization [25], and ASC1, which could be associated with translational repression [26]. Particularly, actin participates in the asymmetric distribution of damaged proteins between mother and daughter cells [25,27]. Aβwt-GFP also shows upregulation of sugar metabolism, including energy production (e.g. FBA1, TPI1) [28], and amino acid metabolism (MET6), which could be related to the enhanced protein turnover. These findings together with the inclusion body recruitment process [15] (figure 1) resemble the foci formation of a thermolabile variant of UBC9—an active and energy supported process associated with PQC (figure 3) [29]. In agreement, as happens for UBC9, ATP depletion results in foci recruitment problems (electronic supplementary material, figure S4).

The proteins upregulated in Aβm-GFP are primarily involved in oxidation, ion transport and translation (electronic supplementary material, table S4). The resultant differences at the level of redox processes could be caused by oxidative stress. Actually, misfolded proteins could trigger oxidative stress by interfering with chaperones that assist in the folding of mitochondrial proteins [12]. Given that Aβm-GFP is present throughout the cytoplasm and at higher concentration than Aβwt-GFP, it is reasonable to imagine that more transient and harmful interactions could occur in this scenario [30,31], facilitating the emergence of misfolding and oxidative events (figure 3). In fact, after 20 h of expression, Aβm-GFP presents higher oxidative levels than Aβwt-GFP (electronic supplementary material, figure S4). Importantly, our data raise the intriguing possibility that under stress some proteins coalesce into large aggregates, whereas others remain distributed in the cytoplasm, despite being mostly insoluble, with implications for physiological wellbeing. The threshold of aggregation propensity could be associated with a tolerable oxidation level above which cells will actively recruit misfolded proteins into foci. This action will facilitate removal of misfolded proteins and reduce the toxic load but bring with it an energetic cost (figure 3). Actually, decreasing Aβ concentration reduces oxidative stress, which, in turn, improves memory in an Alzheimer's disease mouse model [32].

## 4. Concluding remarks

We therefore suggest that, despite its generic nature, the cellular response to misfolding is adaptable and might be a protective mechanism to minimize damage owing to oxidative stress. As Escusa-Toret *et al.* [29] recently suggested, protein sequestration into inclusion bodies occurs not only when quality control machinery fails but, as we observed here, potentially as part of the UPR to deal with dangerous misfolded proteins [29]. This stratagem allows the cell to distinguish inherently toxic proteins to most efficiently manage limited bioenergetics and homeostatic resources. According to this hypothesis, protein foci formation may serve as a protective mechanism and the energetic cost associated with it would be spent only when the toxic risk would exceed a tolerance threshold. Future research should now investigate how the cell recognizes which specific protein species need to be recruited into aggregates [27].

## 5. Material and methods

### 5.1. Strains and culture conditions

The MC1061 *E. coli* strain (araD139 Δ(araA-leu)7697 ΔlacX74 galK16 galS15(GalS) λ-e14-mcrA0 relA1 rpsL150(strR) spoT1 mcrB1 hsdR2) was employed to amplify the shuttle vector pESC-URA (Agilent Technologies) carrying an empty plasmid or the desired GFP fusion [15] (electronic supplementary material, table S6)*.* BY4741 *Saccharomyces cerevisiae* strain (*MATa*; *his3*Δ1; *leu2Δ0*; *met15Δ0*; *ura3Δ0*) was employed as a model to express the desired proteins. BY4741, with mCherry constitutively expressed encoded in the genome, was employed as a control strain for the growth rate measurement. The cells were transformed following the lithium acetate method. All yeast cultures studied started from fresh transformed colonies, after 3 days of growing on synthetic solid media deficient in uracil (SC-URA). The cultures were grown as described in Morell *et al.* [15]. Briefly, a saturated overnight culture grown in SC-URA containing raffinose was employed to inoculate a culture of SC-URA with galactose (induction media) at an OD_600_ of 0.02. This culture was incubated at 30°C for 9 h to increase the existence of multiple aggregates in the cells (figure 1*a,b*). Before performing an assay, the cells were observed under a fluorescent microscope (LSM710, Zeiss) to monitor the formation of intracellular aggregates.

### 5.2. mRNA expression levels

Yeast cells were lysed by incubating them in 0.2 M lithium acetate, 1% SDS solution. Briefly, 10 ml of cells grown with 2% galactose for 9 h was centrifuged and suspended in 1 ml of 0.2 M lithium acetate, 1% SDS solution. After 5 min at 70°C, 3 ml of TRIzol was added. The RNA was extracted with TRIzol reagent following the instructions provided by Life Technologies. Retrotranscription was performed using the RevertAid H Minus First Strand cDNA Synthesis Kit (Thermo Scientific) and the random hexamer primers included with the kit. The concentration of the cDNA generated was adjusted and qPCR was performed using SYBR Green PCR Master Mix (Life Technologies). The concentration of RNA purified was measured before the retrotranscription. We employed Primer-BLAST to design the primers for the qPCR to ensure that they did not bind any yeast sequence and amplify a region of 97 bp located at the 3′ region of GFP (electronic supplementary material, table S6). The reactions were performed in an Eco Illumina qPCR (Illumina). The mRNA of three different reference genes (*TAF10*, *TFC1* and *UBC6*) was measured and its arithmetic mean employed to normalize the data. The primers to amplify the reference genes were obtained from Teste *et al.* [33]. The mRNA quantification was measured by the ΔCt method. The variation between Aβwt-GFP and Aβm-GFP was measured as ΔΔCt.

### 5.3. Western blotting

Yeast cultures were grown for 9 h in induction media. Culture (20 ml) was divided in two and each 10 ml was centrifuged. One pellet was employed to measure the total fraction, for which it was resuspended in 75 μl of lysis buffer (10 mM Tris–HCl pH = 8, 150 mM NaCl, 0.05% Tween 20, 10% glycerol, 5 mM EDTA, 1 mM DTT, 2 mM PMSF) and 25 μl NuPAGE LDS sample buffer with 2.5% 2-mercaptoethanol (w/w) and incubated at 100°C for 5 min. The other pellet was resuspended in 75 µl Y-Per yeast protein extraction reagent (Thermo Scientific) supplemented with 0.1 mM PMSF and incubated at room temperature with agitation for 20 min. Then, the sample was centrifuged to separate the soluble (supernatant) and insoluble (pellet) fractions. The insoluble fraction was resuspended again in 75 μl of PBS; 25 μl of NuPAGE LDS sample buffer with 2.5% 2-mercaptoethanol (w/w) was added to both fractions, which were then incubated at 100°C. To separate the proteins, 5 μl of the total fraction and 10 μl of soluble or insoluble fractions were eluted into a 12% acrylamide bis–tris NuPAGE gel. The proteins were transferred onto a nitrocellulose membrane. The total fraction of a yeast strain expressing GFP alone was employed as a positive control. The antibodies employed were: MCA78G anti-tubulin alpha (Abd Serotec) from rat, 5204–2504 goat antirat (Abd Serotec), A-6455 anti-GFP (Life Technologies) from rabbit, anti-rabbit A9169 (Sigma) from goat.

ECL Western blotting detection reagents (GE Healthcare Life Sciences) were employed to detect the GFP and tubulin alpha bands. Images were obtained with a Gel Doc XR ChemiDoc, and the bands quantified employing the volume tools of the ImageLab (4.0) software. The tubulin alpha bands were employed to normalize the GFP intensity.

### 5.4. *In vivo* half-life measurement

Yeast cells were grown with 2% galactose for 9 h. Protein production was then stopped by adding 35 μg ml^−1^ of cycloheximide. At different times (0, 30, 60, 90, 240, 480 and 1440 min) an aliquot of culture was taken. Each sample was centrifuged and suspended in PBS. The samples were vortexed for 1 min before measuring the fluorescence loss using a BD LSR II flow cytometer system (BD Biosciences). Cells were counted at a maximum flow rate of 600 events per second. GFP fluorescence was measured using a 488 nm laser for excitation and a 525/50 nm band pass filter. The fluorescence loss was measured as the number of fluorescent cells at every time point. To calculate the ratio of fluorescence loss, the data were fitted to a one phase decay curve with GraphPad Prism 5 software (GraphPad Software).

### 5.5. Cell growth rate measurement

Aβwt-GFP and Aβm-GFP were grown in competition against a control strain encoding for mCherry and carrying an empty pESC-URA vector. To perform this competition, yeast cells were grown in SC-URA containing raffinose overnight. The cells were inoculated into SC-URA raffinose media and grown for 3 h until achieving exponential phase. These cultures were adjusted to the same concentration. To start the competition experiment, the same proportion of mCherry strain and Aβwt-GFP or Aβm-GFP was inoculated into SC-URA galactose media. The culture densities were controlled to preserve cells at exponential growth by not surpassing an OD_600_ of 0.7. Samples were collected at 6, 21, 29, 45, 53 and 70 h. The samples were centrifuged, suspended in PBS and vortexed for 1 min before measurement of the proportion of red and green fluorescent cells. We used a BD LSR II flow cytometer system (BD Biosciences) with a maximum flow rate of 600 events per second. A 488 nm excitation laser and a 525/50 nm band pass filter were employed to analyse GFP fluorescence and mCherry was measured with a 561 nm laser and 610/20 filter. The ratio between the control (mCherry) and Aβ-GFP cells was calculated and plotted against the number of generations of the control strain (electronic supplementary material, figure S3). An *F*-test was applied on the Aβ cells/control ratios to measure the difference between Aβwt-GFP or Aβm-GFP slopes.

### 5.6. Oxidative stress and protein aggregation disruption

Aβwt-GFP and Aβm-GFP cells were grown overnight (16 h) in SC-URA and galactose and then inoculated in fresh media for 4 h to achieve exponential phase. The culture was then centrifuged and resuspended in PBS containing 10 μM dihydroethidium (Life Technologies, D23107). The culture was incubated in the dark for 10 min and then washed twice in PBS before image acquisition. To test the effect of ATP depletion on foci formation, Aβwt-GFP was grown for 9 h in SC-URA and galactose. The culture was then incubated for 1 h with 10 mM sodium azide and 10 mM deoxyglucose, before being incubated for 10 min with 10 µM of dihydroethidium.

The fluorescence of dihydroethidium was excited at 514 nm and the emission collected between 550 and 700 nm. The GFP fluorescence acquired was excited with a 488 nm laser and the emission collected between 500 and 700 nm as before. ImageJ was used to quantify the dihydroethidium fluorescence intensity for the cells expressing Aβ-GFP. A Kolmogorov–Smirnov test was employed to measure the significance of the data.

### 5.7. Protein sample preparation and CyDye protein labelling

For two-dimensional-DIGE analysis, samples were collected after 9 h of growth. Cells were harvested by centrifugation and the pellets were resuspended with 200 µl of two-dimensional-DIGE labelling buffer (7 M urea, 2 M thiourea, 4% (w/v) CHAPS and 30 mM Tris) containing protease inhibitors. An equal volume of glass beads (0.5 mm from Sigma) was added and shaken in a vortex stirrer at maximum speed for five cycles of 1 min followed by 1 min of cooling on ice. All samples were prepared in parallel. Protein extracts were clarified by centrifugation at 12 000*g* for 10 min at 4°C. The pH of each cell lysate was carefully adjusted to 8.5 with NaOH, and protein concentration was determined using the two-dimensional-Quant kit (GE Healthcare) with BSA as standard. Protein extracts were labelled with the CyDyes (GE Healthcare) prior to electrophoresis. Reconstitution of CyDyes and protein labelling was performed following the manufacturer's instructions. Briefly, proteins were labelled by mixing 240 pmol of fluorochromes with 30 µg of protein and incubated on ice for 30 min in the dark. Lysine (1 µl, 10 mM) was then added to quench the reaction, and the samples were left on ice for 10 min in the dark. A pooled internal standard was performed by mixing 15 µg of each sample. This pool was labelled with Cy2 dye and was included in all gel runs to be used as intragel spot intensity normalization. A dye swap was used between Cy3 and Cy5 to avoid problems associated with preferential labelling. The gels ran simultaneously, with a dye switching between repetitions, plus the internal standard. In the end, 90 μg of proteins (30 µg of each sample) was loaded on each gel and separated by two-dimensional gel electrophoresis.

### 5.8. Two-dimensional gel electrophoresis

For two-dimensional gel electrophoresis, the two samples to be run on the same gel plus the internal standard were mixed before adding 2 × GE lysis buffer (7 M urea, 2 M thiourea, 4% (w/v) CHAPS, 12 µ ml^−1^ DeStreak reagent (GE Healthcare)) and 2% (v/v) ampholytes immobilized pH gradient (IPG) buffer (pH 3–10 NL, GE Healthcare) to a final volume of 125 µl. Isoelectric focusing (IEF) was carried out on pH 3–10 IPG-strips (24 cm, nonlinear gradient; GE Healthcare) using the IPGphor system from GE Healthcare. Immobiline DryStrips were rehydrated overnight with DeStreak rehydration solution (GE Healthcare) before cup-loading of proteins and IEF on an Ettan IPGphor Manifold (GE Healthcare). The migration was performed at 20°C (60 V for 2 h; gradient from 60 to 500 V for 5 h; hold 500 for 1 h, gradient from 500 to 1000 for 3 h; hold 1000 V for 1 h; gradient from 1000 to 8000 V for 4 h, hold 8000 V until 64 000 Vh). After the IEF, IPG strips were equilibrated twice for 15 min in equilibration buffer (50 mM Tris–HCl pH 8.8, 6 M urea, 30% (v/v) glycerol, 2% (w/v) SDS and 0.002% (w/v) bromophenol blue) supplemented with DTT and then with iodoacetamide. Second-dimension SDS–PAGE was performed using 24 cm format 12.5% resolving gel and run at 20°C overnight with 1.5 W per gel, using the Ettan DALT twelve system (GE Healthcare).

### 5.9. Scanning and image analysis

Two-dimensional-DIGE gels were scanned at a pixel size of 100 µm using a Typhoon Imager 9400 (GE Healthcare) at three different wavelengths corresponding to the different CyDyes. Gel images were exported into the Progenesis SameSpot v. 3 image analysis system (Nonlinear Dynamics, UK), where quantitative analysis of protein spots was performed. A total of 1400 protein spots were detected from the 12 gel images analysed (figure 1*e*). Following automatic and subsequent manual editing, aligning and matching procedures as part of the Progenesis SameSpot workflow, ANOVA *p*-values between the samples were calculated within the Progenesis SameSpot software. Variation of protein expression was considered statistically significant if the absolute abundance variation was at least 1.2-fold between spots of any experimental group with a *p* < 0.05 by ANOVA. The spots of interest were visually checked and selected for protein identification by mass spectrometry.

Unsupervised PCA correlation analysis was performed using the statistical tool within the gel analysis software. PCA reduces the complexity of a multidimensional analysis into two principal components, PC1 and PC2, which orthogonally divide the samples based on the two largest sources of variation in the dataset. Clustering of each sample was based on the expression pattern of each spot with a significant ANOVA *p*-value (*p* < 0.05).

### 5.10. Spot handling and protein identification by mass spectrometry

Spots of interest were excised from the gels and proteins subjected to in-gel digestion with trypsin (Promega, Madison, WI). Spots excised, were destained and reduced with dithiothreitol, alkylated with iodoacetamide, and dried in a SpeedVac. Gel pieces were rehydrated with digestion buffer (50 mM NH_4_HCO_3_) containing trypsin (6.7 ng l^−1^; Promega) and incubated overnight at 37°C. The buffered peptides were acidified with formic acid, desalted and concentrated using C8 microcolumns (POROS R2, Applied Biosystems). The peptides were eluted with matrix solution that contained 10 mg ml^−1^ α-cyano-4-hydroxycinnamic acid dissolved in 70% (v/v) acetonitrile/0.1% (v/v) trifluoroacetic acid. The mixture was allowed to air-dry (dried droplet method). Mass spectra were obtained by an Applied Biosystem 4800 Proteomics Analyser (Applied Biosystems, Foster City, CA) in MS and MS/MS mode.

The generated mass spectra were used to search the NCBI protein database with the algorithms Paragon, from ProteinPilot software v. 2.0 (Applied Biosystems, MDS Sciex), and Mowse, from MASCOT-demon v. 2.1.0 Software (Matrix-Science). In the analysis using ProteinPilot, other parameters considered were: enzyme, trypsin; Cys alkylation, iodoacetamide; special factor, urea denaturation; species, none and ID focus, biological modification. All proteins identified by ProteinPilot have a 95% or greater confidence as determined by ProteinPilot unused scores (≥1.3). Regarding MASCOT search, the analysis of results was performed in the GPS Explorer software (Applied Biosystems), using the following parameters: missed cleavage, one; peptide tolerance, 50–75 ppm; fragment mass tolerance, 0.25 Da; fixed modification, carbamidomethylation of cysteine and variable modification, methionine oxidation.

Following these steps, we identified the proteins of 115 spots that comprise 74 unique proteins (electronic supplementary material, tables S1–S2). The molecular mass and isoelectric point determined on the two-dimensional gel of the identified proteins are consistent. There are some proteins that have been identified in more than one spot suggesting the effect of post-translational modifications or protein isoforms. In these cases, the spots with identical protein suffer similar regulation (e.g. spots 68, 1173, 1174 and 1192 identified as MET6 are downregulated in Aβ42wt-GFP cells). In 20 spots, two different proteins were identified and both isoforms are shown in the electronic supplementary material, table S1 (e.g. spot 142 was identified as VMA1 and HSP77).

### 5.11. Gene ontology analysis

The identified proteins were categorized into functional groups using the first entry listed in the gene ontology annotations provided by the KEGG and the cellular component as indicated in the UniProt database (http://www.uniprot.org).

## Supplementary Material

Suplpementary figures and tables
