# Supplementary material for: Proteome response at the edge of protein aggregation
Source: Open Biol. 2015 Feb 11;5(2):140221. doi: 10.1098/rsob.140221 (PMC4345283; doi:10.1098/rsob.140221)
Supplement: Suplpementary figures and tables [file rsob140221supp1.pdf]

Supplementary Information

Proteome response at the edge of protein aggregation

Natália Sanchez de Groot[1*] Ricardo A. Gomes[2], Anna Villar-Pique[3], M. Madan Babu[1], Ana Varela Coelho[2], Salvador Ventura[4*]

1 - Medical Research Council Laboratory of Molecular Biology, Francis Crick Avenue, Cambridge, CB2 0QH United Kingdom
2 - Instituto de Tecnologia Química e Biológica António Xavier, Universidade Nova de Lisboa, Av. da República, 2780-157 Oeiras, Portugal
3 - Department of Neurodegeneration and Restorative Research, University Medical Center Goettingen, Waldweg 33, Goettingen, Germany.
4 - Institut de Biotecnologia i Biomedicina and Departament de Bioquímica i Biologia Molecular, Universitat Autònoma de Barcelona, 08193-Bellaterra (Barcelona), Spain

* Corresponding authors. To whom correspondence should be addressed. E-mail: nsdgroot@mrc-lmb.ac.uk, salvador.ventura@uab.cat

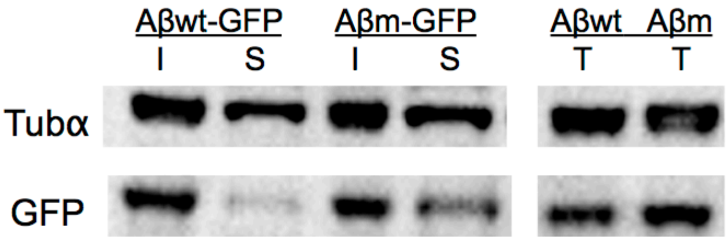

Aβwt-GFP		Aβm-GFP		Aβwt-GFP	Aβm-GFP
Insoluble	Soluble	Insoluble	Soluble	Total	Total
0.61	0.14	0.41	0.34	0.28	0.53

Figure S1. Protein expression and solubility. Analysis of the expression and the soluble/insoluble partition of Aβwt-GFP and Aβm-GFP. The total, soluble and insoluble fractions were separated after 9 hours of induction. Tubulin Alpha (Tubα) was employed to normalise the Aβ-GFP bands.

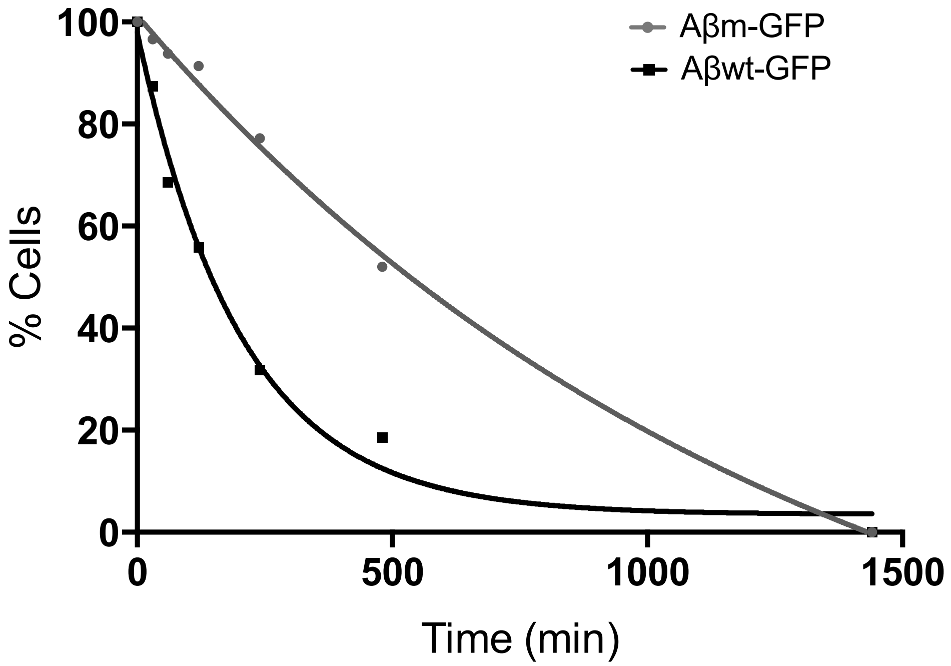

Best-fit values	Aβwt-GFP	Aβm-GFP
K	7.7±1.5•10^{-4} min^{-1}	49±7.0•10^{-4} min^{-1}
Half Life	894±176 min	142±20 min

Figure S2. Protein half-life calculation. Protein production was arrested with cycloheximide and the number of fluorescent cells measured at the times indicated. The number of cells at each time point fitted to an exponential decay. K is the rate constant.

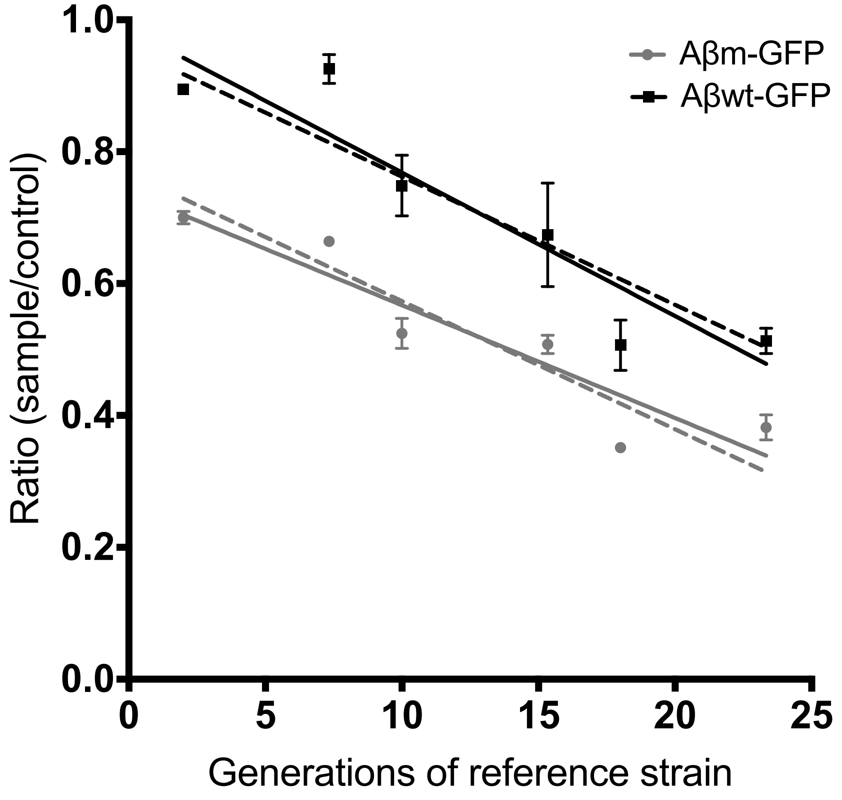

Comparison of Fits	
Null hypothesis	Slope same for all data sets
Alternative hypothesis	Slope different for each data set
P value	0.2279
Conclusion (alpha = 0.05)	Do not reject null hypothesis
Preferred model	Slope same for all data sets
F (DFn, DFd)	1.547 (1,20)

Figure S3. Cell fitness advantage measurement. The ratio between Aβ expressing cells and control cells was measured at the indicated time points. Dashed line represents the common slope displayed by the F-test.

A

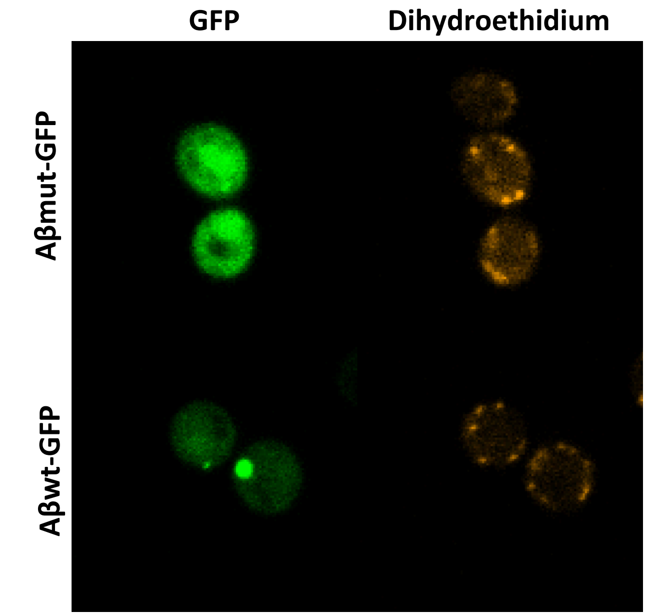

B

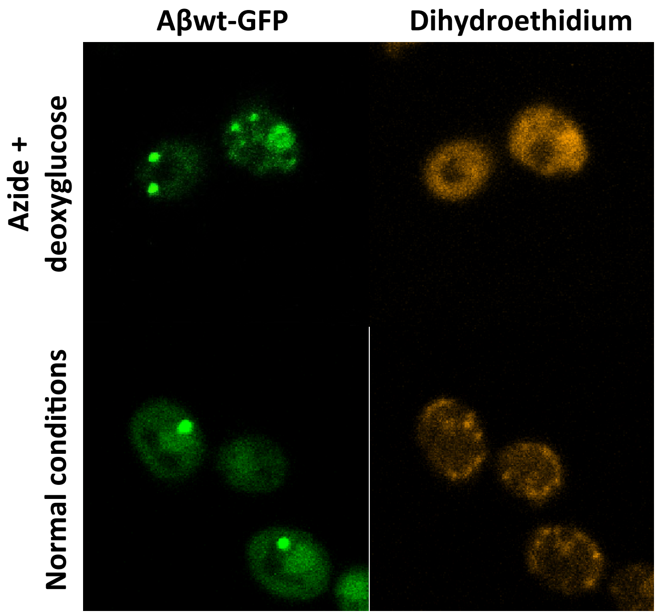

Figure S4. Oxidative stress and protein aggregation disruption. A) Confocal images comparing the oxidative levels between of Aβwt-GFP and Aβmut-GFP after 20h of expression at normal conditions. Average fluorescence intensity (a.u.) per μm^2: Aβwt-GFP=16.72, n=331; Aβmut-GFP=28.22, n=359; $p < 0.0001$. Significance measured with Kolmogorov-Smirnov test. B) Effects on the oxidation levels and the aggregates cohesion due to ATP depletion after 9h of expression.

Table S1. Summary comparing expression, solubility and toxicity of Aβwt-GFP and Aβm-GFP.

	Aβm/Aβwt
**mRNA (ΔCt)**	1.1[a]
**Insoluble**	0.67
**Soluble**	2.43
**Total**	1.89
**Half-life (min)**	6.3
**Growth ratio**	no sig.[b]

a) The variation of mRNA synthesis of Aβwt-GFP and Aβm-GFP was measured as ΔΔCt.
b) The slopes are not significantly different according to F-test (Supplementary Figure 3).

Table S2. Proteins up-regulated when expressing Aβ-GFP

Gene symbol[a]	Protein name	Function[b]	Location[c]	Spot no.	P value	Fold Var.	P. Score[d]
**AI1M**	Putative COX1/OXI3 intron 1	EM	M	570	1.91E-05	1.8	174[e]
**ATP2**	ATP synthase subunit beta	EM	M, C	1044	4.35E-08	2.8	1160
**ATP2**	ATP synthase subunit beta	EM	M, C	1164	4.39E-06	2.3	843
**ATP4**	ATP synthase subunit 4	EM	M	715	3.90E-07	3	350
**ATP7**	ATP synthase subunit d	EM	M	774	1.74E-04	2.6	132
**CDC19**	Pyruvate kinase 1	CM	C	1166	2.22E-05	2.2	469
**COR1**	Cytochrome b-c1 complex subunit 1	EM	M	349	6.00E-03	1.3	463
**CYS3**	Cystathionine gamma-lyase	EM	C, N	453	6.00E-03	1.5	319
**GPM1**	Phosphoglycerate mutase 1	CM	M, C	1216	2.10E-02	1.3	222
**HSC82**	ATP-dependent molecular chaperone HSC82	FSD	M, C	79	6.00E-03	1.5	321[e]
**HSP10**	10 kDa heat shock protein	FSD	M	988	2.80E-02	1.7	228
**HSP60**	Heat shock protein 60	FSD	M, C	1046	4.87E-06	1.8	401
**HSP60**	Heat shock protein 60	FSD	M, C	1048	1.12E-06	1.9	353
**HSP75**	Heat shock protein SSB1	FSD	C	1052	4.06E-04	1.4	302
**HSP77**	Heat shock protein SSC1	FSD	M, N	142	3.00E-03	1.5	182
**HSP77**	Heat shock protein SSC1	FSD	M, N	143	5.24E-04	1.6	166
**HSP77**	Heat shock protein SSC1	FSD	M, N	1052	4.06E-04	1.4	1070
**IPP1**	Inorganic pyrophosphatase	EM	C	562	1.00E-02	1.5	84
**KPYK2**	Pyruvate kinase 2	CM	M	1166	2.22E-05	2.2	77
**MAL31**	Maltose permease MAL31	TP	Mb	247	2.00E-03	1.6	17[e]
**MET30**	F-box protein MET30	FSD	C, N	861	5.00E-03	1.6	437
**MLC1**	Myosin light chain	CD	B	247	2.00E-03	1.6	428[e]
**MLC1**	Myosin light chain 1	CD	B	946	2.00E-03	1.7	166
**MMF1**	Protein MMF1	AM	M	912	1.80E-02	1.4	429
**PGK1**	Phosphoglycerate kinase	CM	M, C	348	9.78E-08	2	906
**POR1**	Mitochondrial outer membrane protein porin 1	ST	M	625	1.90E-07	2.1	658
**POR1**	Mitochondrial outer membrane protein porin 1	ST	M	626	6.29E-09	2.8	871
**POR1**	Mitochondrial outer membrane protein porin 1	ST	M	627	7.50E-06	2.8	59
**POR1**	Mitochondrial outer membrane protein porin 1	ST	M	630	4.83E-05	1.6	282
**URA3**	Orotidine 5'-phosphate decarboxylase	NM	C	630	4.83E-05	1.6	125
**QCR2**	Cytochrome b-c1 complex subunit 2	EM	M	1202	1.01E-05	1.4	510
**QCR7**	Cytochrome b-c1 complex subunit 7	EM	M	907	4.20E-02	1.7	236
**RL22B**	60S ribosomalprotein L22-B	T	C	912	1.80E-02	1.4	64
**RPS12**	40S ribosomal protein S12	T	C	867	4.20E-02	1.6	417[e]
**SLX5**	E3 ubiquitin-protein ligase complex SLX5-SLX8	PM	N	256	2.00E-03	1.8	35
**SOD1**	Superoxide dismutase [Cu-Zn]	TP	M, C	842	1.10E-02	1.5	187
**SOD2**	Superoxide dismutase [Mn]	TP	M	754	6.70E-06	1.6	130
**SOD2**	Superoxide dismutase [Mn]	TP	M	755	3.40E-04	2.5	55
**SSA1**	Heat shock protein SSA1	T	C, CW	133	3.00E-03	1.5	1010
**SSA1**	Heat shock protein SSA1	T	C, CW	143	5.24E-04	1.6	486
**SSA1**	Heat shock protein SSA1	T	C, CW	151	4.41E-04	1.7	714
**SSA1**	Heat shock protein SSA1	T	C, CW	153	2.00E-03	1.7	941
**SSA1**	Heat shock protein SSA1	T	C, CW	1132	3.11E-05	1.8	329
**SSA2**	HeatshockproteinSSA2	T	C, M	151	4.41E-04	1.7	661
**SSA2**	Heat shock protein SSA2	T	C, M	153	2.00E-03	1.7	920
**SSA2**	Heat shock protein SSA2	T	C, M	1132	3.11E-05	1.8	329
**SSZ1**	Ribosome-associated complexsubunitSSZ1	FSD	C, P	1046	4.87E-06	1.8	109
**SSZ1**	Ribosome-associated complex subunit SSZ1	FSD	C, P	1048	1.12E-06	1.9	174
**UBI4**	Ubiquitin (UBI1)	T	C, N	994	2.00E-03	2	193
**VMA1**	V-type proton ATPase catalytic subunit A	EM	V	142	3.00E-03	1.5	238
**VMA4**	V-type proton ATPase subunit	EM	V, Mb	666	6.88E-04	1.5	906
**YNK1**	Nucleoside diphosphate kinase	NM	M, C	861	5.00E-03	1.6	728[e]

a) Standard gene name in the Saccharomyces genome database (www.yeastgenome.org).
b) KEGG function classification: AM - Amino acid metabolism; CD - Cell division; Cmo - Cell motility; CM - Carbohydrate metabolism; EM - Energy metabolism; FSD - Folding sorting and degradation; LM - Lipid Metabolism; NM - Nucleotide metabolism; PM - Protein modification; ST - Signal transduction; T -Translation; TP – Transport; ? - Not classified. Cell metabolism includes: AM, CM, EM, LM and NM.
c) UniProt cellular component: M - Mitochondrion; C - Cytosol; Mb - Membrane; N - Nucleus; B - Cell bud; CW - Cell wall; V - Vacuole; G - Golgi apparatus; ER - Endoplasmic reticulum; R - Ribosome; P - Polysome and Ct - Cytoskeleton.
d) The protein score as given by the GPS Explorer software (Applied Biosystems). All the proteins were identified with at least a 95% confidence.
e) Proteins identified by Protein Pilot. Unused scores are provided to shown that all the proteins were identified with at least a 95% confidence (unused score ≥1.3).

Table S3. Proteins down-regulated when expressing Aβ-GFP

Gene symbol[a]	Protein name	Function[b]	Location[c]	Spot no.	P value	Fold Var.	P. Score[d]
ACB1	Acyl-CoA-binding protein	TP	C, N	1013	1.70E-02	-1.5	146
ACT1	Actin	TP	C, Ct	388	1.00E-02	-2.7	740
ACT1	Actin	TP	C, Ct	389	8.00E-03	-1.9	51
ADH1	Alcohol dehydrogenase 1	CM	C	384	3.40E-02	-1.4	1090
ADH1	Alcohol dehydrogenase 1	CM	C	391	6.00E-03	-1.6	281
ADH1	Alcohol dehydrogenase 1	CM	C	449	8.00E-03	-1.3	636
AHP1	Peroxiredoxin type-2	FSD	C, P	796	1.26E-06	-4.4	267
AHP1	Peroxiredoxin type-2	FSD	C, P	797	9.86E-07	-4.2	469
ALD6	Magnesium-activated aldehyde dehydrogenase	CM	C	193	3.41E-04	-2.7	62
ALD6	Magnesium-activated aldehyde dehydrogenase	CM	C	194	2.84E-05	-3.8	345
ALD6	Magnesium-activated aldehyde dehydrogenase	CM	C	195	1.67E-05	-3.8	2[e]
APT1	Adeninephosphoribosyltransferase1	NM	C, N	1218	5.00E-03	-1.4	114
ARC15	Actin-related protein 2/3 complex subunit 5	Cmo	C, Ct	836	2.50E-02	-1.3	61
ASC1	Guanine nucleotide-binding protein subunit beta-like	T	C	1051	2.00E-03	-1.5	437
ASC1	Guanine nucleotide-binding protein subunit beta-like	T	C	1057	1.90E-02	-1.2	612
CCT8	T-complex protein 1 subunit theta	FSD	C	197	6.32E-05	-2.9	33
CDC48	Cell division control protein 48	FSD	ER	29	1.70E-02	-1.7	717
CPR1	Peptidyl-prolyl cis-trans isomerase	FSD	C	883	3.60E-02	-1.3	436
CPR1	Peptidyl-prolyl cis-trans isomerase	FSD	C	887	1.20E-02	-1.4	212[e]
EFB1	Elongation factor 1-beta	T	R	1061	1.00E-02	-1.5	173
EFB1	Elongation factor 1-beta	T	R	1062	3.20E-02	-1.5	183
ENO2	Enolase 2	CM	M, Mb	325	9.13E-04	-1.8	1290
ENO2	Enolase 2	CM	M, Mb	326	3.10E-02	-1.4	1180
ENO2	Enolase 2	CM	M, Mb	330	1.40E-02	-1.4	1270
ENO2	Enolase 2	CM	M, Mb	429	3.31E-04	-1.7	567
FBA1	Fructose-bisphosphate aldolase	CM	M, C	412	3.00E-03	-1.6	673
FBA1	Fructose-bisphosphate aldolase	CM	M, C	415	3.80E-02	-1.3	282
FBA1	Fructose-bisphosphate aldolase	CM	M, C	418	9.76E-04	-1.8	862
FBA1	Fructose-bisphosphate aldolase	CM	M, C	429	3.31E-04	-1.7	598
GPP1	(DL)-glycerol-3-phosphatase 1	LM	C, N	1050	3.69E-06	-2.1	745
GPP2	(DL)-glycerol-3-phosphatase2	LM	C, N	1050	3.69E-06	-2.1	308
ILV5	Ketol-acidreductoisomerase	AM	M	391	6.00E-03	-1.6	166
MDH1	Malate dehydrogenase	CM	M	541	8.00E-03	-1.6	545
MET6	5-methyltetrahydropteroyltriglutamate-homocysteine	AM	C	68	5.22E-04	-2.1	250
MET6	5-methyltetrahydropteroyltriglutamate-homocysteine	AM	C	1173	2.00E-03	-1.8	322
MET6	5-methyltetrahydropteroyltriglutamate-homocysteine	AM	C	1174	4.30E-04	-2.1	69
MET6	5-methyltetrahydropteroyltriglutamate-homocysteine	AM	C	1192	2.00E-03	-2.1	120
METK1	S-adenosylmethionine synthase 1	AM	C	355	2.00E-03	-1.5	369
MYO1	Myosin-1	CM	B	885	9.00E-03	-2.4	49
PDC1	Pyruvate decarboxylase isozyme 1	CM	C, N	229	9.00E-03	-1.5	717
PDC1	Pyruvate decarboxylase isozyme 1	CM	C, N	239	1.90E-02	-1.5	992
PDC1	Pyruvate decarboxylase isozyme 1	CM	C, N	902	2.10E-02	-2	98
PDC1	Pyruvate decarboxylase isozyme 1	CM	C, N	1156	1.00E-02	-1.4	272
PRO2	Gamma-glutamyl phosphate reductase	AM	C, N	305	7.00E-03	-1.2	147
RPS21A	40S ribosomal protein S21-A	T	C	999	3.40E-02	-1.3	196
RS21B	40S ribosomal protein S21-B	T	C	999	3.40E-02	-1.3	196
SAM2	S-adenosylmethionine synthase 2	AM	?	355	2.00E-03	-1.5	795
SBA1	Co-chaperone protein SBA1	FSD	C, N	1213	3.40E-02	-1.4	136
SEC53	Phosphomannomutase	CM	C	669	3.40E-02	-1.2	61
SEC53	Phosphomannomutase	CM	C	670	4.23E-04	-1.6	2e
SPE3	Spermidine synthase	AM	C, N	585	1.78E-04	-1.5	79
STI1	Heat shock protein STI1	FSD	C	124	2.60E-02	-1.2	824
TOM40	Mitochondrial import receptor subunit TOM40	TP	M, C	381	4.30E-02	-1.6	218
TPI1	Triosephosphate isomerase	CM	M, Mb	686	2.80E-02	-1.2	548
TPI1	Triosephosphate isomerase	CM	M, Mb	705	1.20E-02	-1.5	550
TPI1	Triosephosphate isomerase	CM	M, Mb	1218	5.00E-03	-1.4	564
TRX1	Thioredoxin-1	FSD	C, G	1000	5.00E-03	-1.6	74
TRX2	Thioredoxin-2	FSD	C, G	1000	5.00E-03	-1.6	74
TSA1	Peroxiredoxin TSA1	CM	C	740	3.40E-02	-1.3	260
TSA1	Peroxiredoxin TSA1	CM	C	1217	2.00E-03	-1.6	406
VMA7	V-type proton ATPase subunit	EM	V, Mb	993	4.00E-03	-1.7	66
YDL124W	NADPH-dependent alpha-keto amide reductase	AM	C, N	511	1.50E-02	-1.5	968
YLL066C	Y' element ATP-dependent helicase	?	C	968	3.90E-02	-1.4	17[e]

a) Standard gene name in the Saccharomyces genome database (www.yeastgenome.org).
b) KEGG function classification: AM - Amino acid metabolism; CD - Cell division; Cmo - Cell motility; CM - Carbohydrate metabolism; EM - Energy metabolism; FSD - Folding sorting and degradation; LM - Lipid Metabolism; NM - Nucleotide metabolism; PM - Protein modification; ST - Signal transduction; T -Translation; TP – Transport; ? - Not classified. Cell metabolism includes: AM, CM, EM, LM and NM.
c) UniProt cellular component: M - Mitochondrion; C - Cytosol; Mb - Membrane; N - Nucleus; B - Cell bud; CW - Cell wall; V - Vacuole; G - Golgi apparatus; ER - Endoplasmic reticulum; R - Ribosome; P - Polysome and Ct - Cytoskeleton.
d) The protein score as given by the GPS Explorer software (Applied Biosystems). All the proteins were identified with at least a 95% confidence.
e) Proteins identified by Protein Pilot. Unused scores are provided to shown that all the proteins were identified with at least a 95% confidence (unused score ≥1.3).

Table S4. Proteins with different levels between Aβ42wt-GFP and Aβm-GFP

Gene symbol[a]	Protein name	Function[b]	Location[c]	Spot no.	P value	Fold Var.	P. Score[d]
**FBA1[g]**	Fructose-bisphosphate aldolase	CM	M, C	412	7.00E-03	1.3	412
**MET6[g]**	5-methyltetrahydropteroyltriglutamate	AM	C, Mb	1173	2.00E-03	1.8	322
**MET6[g]**	5-methyltetrahydropteroyltriglutamate	AM	C, Mb	1174	4.30E-04	2.1	69
**SEC53[g]**	Phosphomannomutase	CM	C	670	8.00E-03	1.3	2e
**TPI1[g]**	Triose phosphate isomerase	CM	M	705	4.30E-02	1.3	550
**URA3[g]**	Orotidine 5'-phosphate decarboxylase	N	C	631	1.00E-02	1.4	874
**URA3[g]**	Orotidine 5'-phosphate decarboxylase	N	C	640	9.00E-03	1.5	808
**ARC15[f,g]**	Actin-related protein 2/3 complex subunit 5	Cmo	C, Ct	836	2.50E-02	-1.3	61
**ASC1[f,g]**	Guanine nucleotide-binding protein subunit beta-like	T	C	1057	1.90E-02	-1.2	612
**ATP2[h]**	ATP synthase subunit beta	EM	M	1044	4.35E-08	2.8	1160
**RPS12[h]**	40S ribosomal protein S12	T	C, R	867	2.57E-04	1.6	117
**SOD2[h]**	Superoxide dismutase [Mn]	TP	M	755	1.70E-02	1.5	55
**TDH3[h]**	Glyceraldehyde-3-phosphate dehydrogenase 3	CM	M, C	726	8.00E-03	1.2	125
**AI1M[f,h]**	Putative COX1/OXI3 intron 1	EM	M	570	1.91E-05	1.8	174e
**IPP1[f,h]**	Inorganic pyrophosphatase	EM	C	562	1.00E-02	1.5	84

Proteins with statistically different spot volume when comparing only Aβ42wt-GFP and Aβm-GFP.
a) Standard gene name in the Saccharomyces genome database (www.yeastgenome.org).
b) KEGG function classification: AM - Amino acid metabolism; CD - Cell division; Cmo - Cell motility; CM - Carbohydrate metabolism; EM - Energy metabolism; FSD - Folding sorting and degradation; LM - Lipid Metabolism; NM - Nucleotide metabolism; PM - Protein modification; ST - Signal transduction; T -Translation; TP – Transport; ? - Not classified. Cell metabolism includes: AM, CM, EM, LM and NM.
c) UniProt cellular component: M - Mitochondrion; C - Cytosol; Mb - Membrane; N - Nucleus; B - Cell bud; CW - Cell wall; V - Vacuole; G - Golgi apparatus; ER - Endoplasmic reticulum; R - Ribosome; P - Polysome and Ct - Cytoskeleton.
d) The protein score as given by the GPS Explorer software (Applied Biosystems). All the proteins were identified with at least a 95% confidence.
e) Proteins identified by Protein Pilot. Unused scores are provided to shown that all the proteins were identified with at least a 95% confidence (unused score ≥1.3).
f) Protein that does not follow the two main expression trends when comparing with the control strain (Figure 1, Table S2 and S3).
g) Proteins up-regulated or down-regulated in Aβ42wt-GFP.
h) Proteins up-regulated in Aβm-GFP.

Table S5. Common proteins associated to UPR-Cyto

Proteins detected at 6	Proteins detected at 15
AHP1	ADH1
EFB1	ASC1
ENO2	ATP2
HSC82	CPR1
HSP60	DPS1
ILV5	EFB1
PGK1	ENO2
SSA1	FBA1
SSA2	GPP1
SSC1	HSP10
STI1	HSP60
	ILV5
	NDK1
	PDC1
	PGK1
	POR1
	PYK1
	RPS12
	SAM2
	SOD1
	SSA1
	SSA2
	SSB1
	SSC1
	URA3

List of proteins shared between the present work, Geiler-Samerotte *et al.* and Gomes *et al*. . These are proteins that change their expression levels as a response to a cytosolic unfolded stress.

6. Geiler-Samerotte KA et al (2011) Proc Natl Acad Sci U S A 108: 680-685
15. Gomes RA et al (2012) PLoS One 7: e50123

Table S6. List of plasmids and primers used in this study.

Name	Sequence/Source
pESC-URA	Agilent Technologies
pESC-URA Aβwt-GFP	Morell *et al.*
pESC-URA Aβm-GFP	Morell *et al.*
Primer forward (3' GFP)	CTGTCGACACAATCTGCCCT
Primer reverse (3' GFP)	TGCCATGTGTAATCCCAGCA
Primers F/R TAF10	Teste *et al.*
Primers F/R TFC1	Teste *et al.*
Primers F/R UBC6	Teste *et al.*
